# Effects of occipital-atlas stabilization in the upper cervical spine kinematics: an in vitro study

**DOI:** 10.1038/s41598-021-90052-6

**Published:** 2021-05-25

**Authors:** César Hidalgo-García, Ana I. Lorente, Carlos López-de-Celis, Orosia Lucha-López, Miguel Malo-Urriés, Jacobo Rodríguez-Sanz, Mario Maza-Frechín, José Miguel Tricás-Moreno, John Krauss, Albert Pérez-Bellmunt

**Affiliations:** 1 Unidad de Investigación en Fisioterapia, Facultad de Ciencias de la Salud de la Universidad de Zaragoza, c/ Domingo Miral s/n, 50009 Zaragoza, Spain; 2 Impact Laboratory, Aragon Institute of Engineering Research, Universidad de Zaragoza, Alcañiz, Spain; 3 ACTIUM Functional Anatomy Group, Universitat Internacional de Catalunya, Barcelona, Spain; 4 School of Health Sciences, Oakland University, Rochester, MI USA

**Keywords:** Anatomy, Medical research

## Abstract

This study compares upper cervical spine range of motion (ROM) in the three cardinal planes before and after occiput-atlas (C0–C1) stabilization. After the dissection of the superficial structures to the alar ligament and the fixation of C2, ten cryopreserved upper cervical columns were manually mobilized in the three cardinal planes of movement without and with a screw stabilization of C0–C1. Upper cervical ROM and mobilization force were measured using the Vicon motion capture system and a load cell respectively. The ROM without C0–C1 stabilization was 19.8° ± 5.2° in flexion and 14.3° ± 7.7° in extension. With stabilization, the ROM was 11.5° ± 4.3° and 6.6° ± 3.5°, respectively. The ROM without C0–C1 stabilization was 4.7° ± 2.3° in right lateral flexion and 5.6° ± 3.2° in left lateral flexion. With stabilization, the ROM was 2.3° ± 1.4° and 2.3° ± 1.2°, respectively. The ROM without C0–C1 stabilization was 33.9° ± 6.7° in right rotation and 28.0° ± 6.9° in left rotation. With stabilization, the ROM was 28.5° ± 7.0° and 23.7° ± 8.5° respectively. Stabilization of C0–C1 reduced the upper cervical ROM by 46.9% in the sagittal plane, 55.3% in the frontal plane, and 15.6% in the transverse plane. Also, the resistance to movement during upper cervical mobilization increased following C0–C1 stabilization.

## Introduction

The occipital-atlas (C0–C1) and atlas-axis (C1–C2) segments join the head to the most mobile region of the spine. The lack of intervertebral discs, the horizontal nature of the joints, and the specialized muscles and ligaments of these segments produce complex three-dimensional kinematics^1^. Due to the complex kinematics within the upper cervical spine, it has been suggested that it be considered as one functional unit, especially in axial rotation. Bogduk and Mercer (2000) proposed that C0–C1 moves primarily in flexion–extension, while C1–C2 mainly rotates. However, Bogduk and Mercer’s findings suggest that interactions between C0–C1 and C1–C2 vary depending on the specific planes of movement^2^.

In the sagittal plane, most studies agree that the average motion for C0–C1 and C1–C2 is 14–15° and 10–21°, respectively^2,3^. However, within the literature, there seems to be variability in the specific contributions of C0–C1 versus C1–C2 during sagittal plane movements. Chancey et al. (2007) described that 41–45% of the upper cervical flexion and 69–71% of the extension occurred in C0–C1^4^, Fujimori et al. (2013) concluded that C0–C1 works mainly for flexion-extension^5^ and Bogduk and Mercer (2000) stated that C0–C1 facilitates C1–C2 motion and that C1–C2 is moved passively by forces coming from C0–C1^2^.

Upper cervical spine range of motion (ROM) in the frontal plane is very limited^6^. Bogduk and Mercer (2000) concluded that C0-C2 move and function as one unit^2^. Limitations in side bending ROM at C1–C2 is thought to be caused by contralateral alar ligament tension or by the impaction of the lateral mass of atlas on the odontoid process^7^. Osmotherly et al. (2012) concluded that any lateral flexion movement of the upper cervical spine (UCS), when C2 is stabilized, is a sign of craniocervical instability^8^.

Approximately 60% of the total cervical ROM in the transverse plane is produced by the UCS^9^. Salem et al. (2013) indicated that C1–C2 shows the largest magnitude of axial rotation with a minimal contribution from C0–C1^10^. Kang et al. (2019) commented that axial ROM at C0–C1 has rarely been examined in cadaver studies^9^. In fact, several authors have disregarded C0–C1 motion completely when studying upper cervical axial rotation^11–14^.

However small the actual motion occurring during rotation at C0–C1, there is an emerging body of evidence supporting the notion that C0–C1 plays a relevant role in the rotation ROM at C1–C2. Improvement of C1–C2 rotation has been demonstrated following the application of manual therapy in the form of translatoric mobilization to C0–C1 in participants with restricted UCS axial rotation^15^, patients with cervicogenic headache^16^, and patients experiencing chronic cervicalgia^17–19^. This approach is based on a rationale that restricted mobility of the C0–C1 segment could limit C1–C2 movement during rotation due to the alar ligament connection across each joint^20,21^. The purpose of this study is to compare upper cervical ROM in the three cardinal planes before and after C0–C1 stabilization using an in vitro design.

## Methods

### Sample

Ten cervical spines and heads from cryopreserved cadavers (9 males, 1 female, mean age: 74 years, range 63–85 years) were examined. All specimens were visually checked for any anatomical condition that would influence ROM. In addition, all samples were required to be free of any disease or contamination. All specimens were donated to Universitat Internacional de Catalunya. Informed consent was obtained from a next of kin and/or legal guardian of the cadaver. The study was approved by a Research Ethics Committee from UIC-Barcelona (Ref. CBAS-2017-03) and all methods were carried out in accordance with relevant guidelines and regulations.

### Anatomical and biomechanical procedure

This study examines the kinematic behavior of the upper cervical spine during movements of the head in the three cardinal planes before and following a screw stabilization at C0–C1.

**Figure 1:**
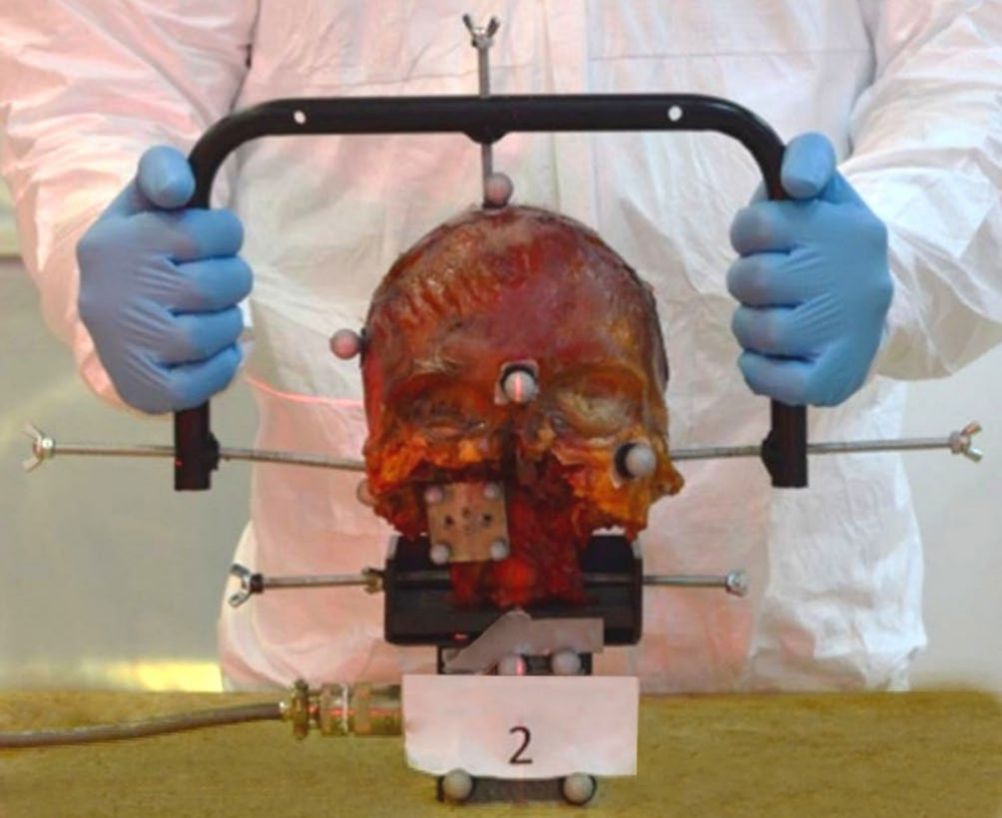
C0-C2 specimen: starting set up of the test.

All specimens were stored at − 14 °C and thawed to room temperature 24 h before testing. The preparation procedure was as follows: First, all spinal segments caudal to C2 vertebra were removed by disarticulating C2–C3 by cutting through the intervertebral disc and zygapophysial facet joint capsules. Second, all muscle tissue was removed without disrupting ligamentous tissues. Third, the cranial posterior third of the skull was removed^22^ to extract the brain and visualize the foramen magnum. The integrity of the posterior arch of atlas was maintained. Forth, the brainstem, spinal cord, dura, and part of the tectorial membrane were removed to expose the alar ligament. Finally, to allow the attachment of the measurement sensors, the mandible, and upper maxilla were removed. Afterward, a metallic handlebar was attached to the skull by three points: one in each auditory canal and one at the top of the head (Fig. 1). The handlebar was designed to move the head without contacting any attached sensors.

The C2 vertebra was then fixed to the load cell (MC3-6-100 Force and Torque Sensor, Advanced Mechanical Technology Inc., Watertown, USA), which measured the torque required to generate the movement in the three cardinal planes. The C2 vertebra was screwed to a metallic support, which was attached to the load cell. The specimen was kept in an upright position (head on top and C2 below) and the three anatomical planes were aligned with the three axes of the load cell. The tester moved the skull from the posterior part of the specimen (Fig. 1).

The force applied by the tester when moving the specimens was converted to newtons from the torque measured by the load cell. The distances between the handlebar and the estimated axes of rotation were used for this calculation: 130 mm for flexion–extension and lateral flexion movements (the height between the center of the hands and the mid-height of C2, as both hands were at the same level), and 150 mm for rotation (the half of the metallic handlebar width). These two measurements were approximated to those two values due to the small variations shown by the instantaneous centres of rotation within individual segments^2,23^. Therefore, the values reported in newtons represent the total load from both hands of the tester in the main plane of the motion.

C2 and the head were aligned in the mid position before each test. To find the mid position, a Frankfurt horizontal plane, which can be considered the physiologic horizontal reference^24^, was laterally marked on the head (through the external auditory meati and the infraorbital foraminae), and a vertical line was marked on the center of the face. This vertical line was a straight up mark from the centre of the chin to the centre of the forehead, running through the centre of the nose. These two markings were aligned with references given by two red light lasers calibrated to the horizontal and vertical.

An optical motion capture system (TS Series, Vicon, Oxford, UK) of four cameras tracked the motion of the head, C1, and C2. The measurement error of this system is 0.0130°, therefore the motions are described with one significant digit. Retroreflective spherical markers were directly placed on the head with glue (Loctite Super Glue-3, Henkel, Germany) (Fig. 1). For C1 a total of four markers were attached on a metallic plate, which was screwed to the vertebra (two parker screws of 8 mm). The plate and its markers were positioned so that there was no interference with the motion between C1 and the skull or C2. The C1 motion was tracked to assess the C0–C1 motion pre- and post-screw fixation. Finally, for C2, markers were fixed on the load cell attached to the C2 vertebra.

To calculate the local coordinate systems of the head, C1, and C2, a 3D measuring device (FaroArm, FARO Technologies, Lake Mary, FL, USA) was installed near the load cell, and anatomical landmarks were measured on the (1) skull: right and left auditory meati and right infraorbital foraminae, (2) C1: symmetrical right and left landmarks on the transverse processes, anterior and posterior tubercles, and (3) C2: symmetrical right and left landmarks on the transverse processes, lowest anterior central point on the body, and lowest central point on the spinous process. Using these landmarks for each segment, the coordinate systems had the X-axis pointing forward, the Y-axis pointing from left to right, and completing a right-hand-oriented coordinate system, the Z-axis pointed downwards. By using both the Vicon system and FaroArm it was possible to measure the motion of each segment. The equations required to define the local coordinate systems can be found in Slykhouse et al. (2019)^25^. The coordinate transformation between FaroArm, the optical markers, and the bones has been previously described in detail by Shaw et al. (2009)^26^.

Synchronized data collection from both the load cell and motion capture systems, was made possible by the installation of a manual trigger that started both systems simultaneously. Both records ended after a pre-defined time of 15 or 20 s, depending on the movement. To compare the motion among all the specimens, the motion was measured at four different instances with the same load: 1 N, 2 N, 3 N and 4 N. Additionally, the maximum load applied and the maximum range of motion was also analyzed.

**Figure 2:**
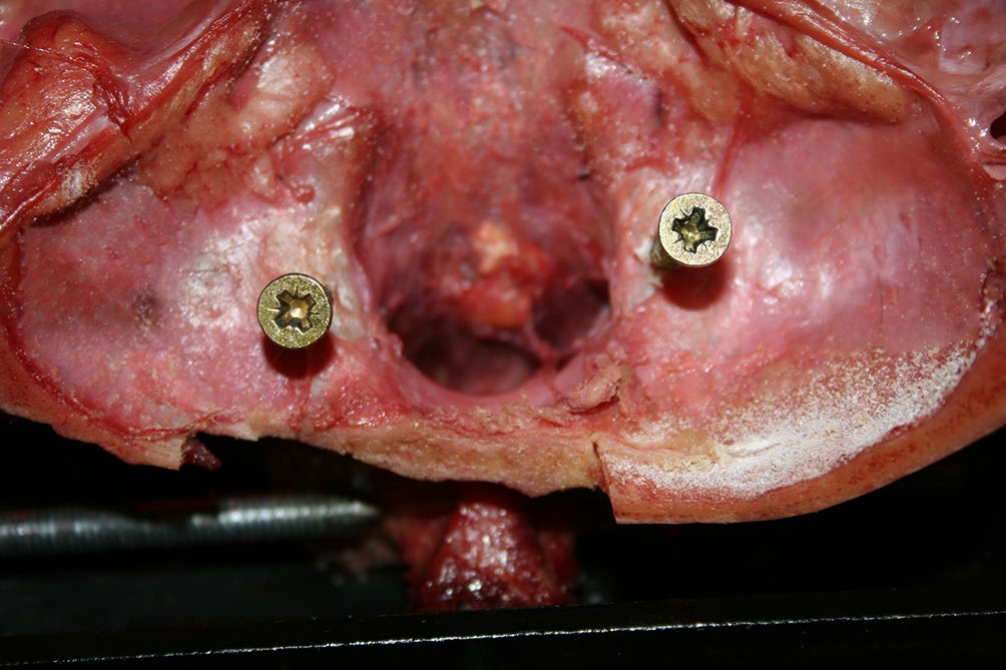
Screw stabilization of occipital-atlas (C0–C1) segment.

Specimens were moved in each plane four times, and always in the same order starting from the neutral position: flexion–extension, right-left lateral flexion, and right-left axial rotation. The first two motions were used as a warm-up to reduce the influence of soft tissue viscoelasticity^27^. Measurements were recorded on the third (prior to C0–C1 stabilization) and forth (post C0–C1 stabilization). All pre-C0–C1 stabilization movements were performed first; then, the post-C0–C1 stabilization movements were performed using the order indicated at the beginning of the paragraph. For the C0–C1 screw stabilization, the occipital entry point of the screw was approximately 5 mm lateral to the foramen magnum, pointing in the direction of and penetrating into the lateral mass of atlas (Fig. 2). The adequacy of screw placement was monitored visually and the C0–C1 and C1–C2 mobility was checked after the screw placement. Approximately 10 mm of the unthreaded portion of the screw remained protruded following screw fixation. All movements were induced manually until a marked resistance was perceived by the tester, a researcher with more than 15 years of clinical experience treating patients with upper cervical impairments who was also a credentialed manual therapist. To prevent dehydration and ensure physiological viability, the dissection room was maintained with a temperature between 17.0° and 17.8° Celsius, and a humidity between 47 and 52%.

Statistical analysis was conducted using SPSS 23.0 (IBM, Armonk, New York). The mean and standard deviation were calculated for each variable. A Wilcoxon Signed Rank Test was performed to analyze intergroup differences, with a significance level set at *p* < 0.05.

### Ethical approval

Research Ethics Committee from UIC-Barcelona. Ref. CBAS-2017-03.

## Results

Table 1 shows the minimum and maximum segmental motion measured for C0–C1 and C1–C2 in the cardinal planes without and with C0–C1 stabilization. Following stabilization, movement between occipital and atlas was reduced by 74.6%, 76.8%, and 90.9% in the sagittal, frontal, and transverse planes, respectively.

**Table 1:** Minimum and maximum intervertebral motion (in degrees) for C0–C1 and C1–C2 in the cardinal planes in both conditions: normal and C0–C1 stabilization (C0–C1 stab).

Movement	Normal (degrees)		C0C1 stab (degrees)		C0–C1 Movement restriction with C0–C1 stab
	C0–C1	C1–C2	C0–C1	C1–C2	
Flexion	− 5.8 to 15.2	2.8–23.5	− 0.9 to 6.2	3.7–15.5	74.4%
Extension	2.5–20.8	− 0.4 to 8	0.5–9	0.7–10.2	
Lateral flex.—right	0.9–8.8	0.4–4	− 0.1 to 1.2	− 0.2 to 4.2	76.9%
Lateral flex.—left	1.7–6.7	0.2–5.2	0.3–2.6	0.2–4.1	
Rotation—right	− 2.1 to 6.7	21.9–42.9	− 5.8 to 5.7	19.4–42.1	90.9%
Rotation—left	− 0.9 to 10.4	13.5–37.6	− 3.4 to 3.4	13.1–35.2	

These are the values for two specimens in each condition, and the rest of the specimens showed values between this range. The percentages show the C0–C1 movement restriction with C0–C1 stabilization.

### Upper cervical sagittal plane mobility

**Figure 3:**
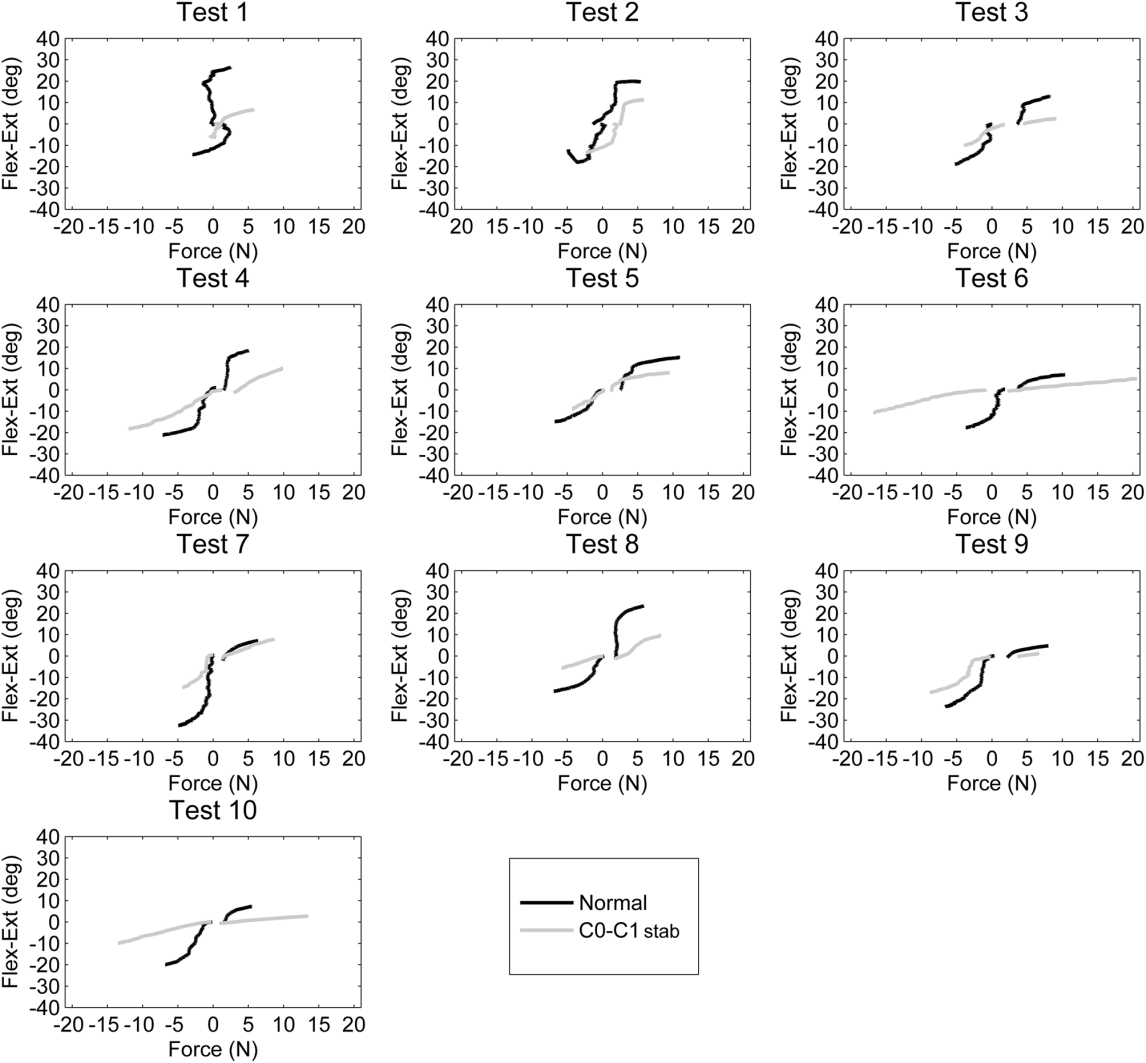
Forces required for flexion (negative values) and extension (positive values) during the full range of motion in the 10 specimens: normal and with C0–C1 stabilization.

Figure 3 illustrates the amount of force applied and the resultant flexion and extension movement for all ten specimens without (illustrated in black) and with C0–C1 stabilization (illustrated in grey). Positive values indicate extension, and negative values indicate flexion. Table 2 contains the angles recorded for each specimen when the applied forces were 1 N, 2 N, 3 N, and 4 N, as well as the force applied to achieve maximum ROM with non-stabilized and stabilized C0–C1 configurations.

**Table 2:** Flexion–extension in degrees for the force values of 1, 2, 3, and 4 N during the motion, and the maximum force (F. Max) with its range of motion (ROM Max).

Test		Flexion (degrees)						Extension (degrees)					
		Force					ROM Max	Force					ROM Max
		1 N	2 N	3 N	4 N	F. Max		1 N	2 N	3 N	4 N	F. Max	
1	Normal	12.7	13.8			2.9	14.5	25.1	26.0			2.5	26.6
	C0C1 stab					0.5	6.2		3.2	4.5	5.4	5.8	6.8
	Difference					− 2.4	− 8.3		− 22.8			3.3	− 19.8
2	Normal	6.5	15.2	17.6	16.6	4.9	17.9	5.8	18.7	19.7	19.9	5.4	20.0
	C0C1 stab	11.9	13.2			2.4	13.4			8.5	10.4	5.9	11.3
	Difference	5.4	− 2.0			− 2.5	− 4.5			− 11.2	− 9.5	0.5	− 8.7
3	Normal	9.0	13.4	15.3	16.9	5.3	18.9				1.9	8.2	12.9
	C0C1 stab	3.9	7.4	9.2		4.0	10.1					9.1	2.5
	Difference	− 5.1	− 6.0	− 6.1		− 1.3	− 8.8					0.9	− 10.4
4	Normal	3.2	11.7	18.1	18.9	7.1	21.2		8.9	16.1	17.4	5.0	18.5
	C0C1 stab	2.7	4.4	7.0	8.3	12.0	18.4				0.9	9.9	10.1
	Difference	− 0.5	− 7.3	− 11.1	− 10.6	4.9	− 2.8				− 16.5	4.9	− 8.4
5	Normal	2.5	7.6	10.2	12.0	6.8	14.9			5.4	8.1	11.0	15.2
	C0C1 stab	2.9	5.8	6.7	8.6	4.3	9.0		3.0	4.9	5.6	9.5	8.0
	Difference	0.4	− 1.8	− 3.5	− 3.4	− 2.5	− 5.9			− 0.5	− 2.5	− 1.5	− 7.2
6	Normal	14.4	16.0	17.2		3.7	17.7				1.8	10.4	7.0
	C0C1 stab	0.1	0.3	0.5	1.0	16.7	10.8					20.6	5.3
	Difference	− 14.3	− 15.7	− 16.7		13.0	− 6.9					10.2	− 1.7
7	Normal	21.0	27.4	29.4	31.8	4.9	32.4		1.0	3.7	5.2	6.4	7.3
	C0C1 stab	6.6	10.1	12.2	14.2	4.3	14.8		0.5	1.5	2.7	8.7	7.9
	Difference	− 14.4	− 17.3	− 17.2	− 17.6	− 0.6	− 17.6		− 0.5	− 2.2	− 2.5	2.3	0.6
8	Normal	4.6	10.6	12.9	14.3	6.9	16.5		15.9	20.1	21.7	5.8	23.7
	C0C1 stab	0.6	1.8	2.9	3.9	5.8	5.7			0.3	2.2	8.3	9.5
	Difference	− 4.0	− 8.8	− 10.0	− 10.4	− 1.1	− 10.8			− 19.8	− 19.5	2.5	− 14.2
9	Normal	2.1	14.3	16.3	19.7	6.6	23.7			1.6	2.7	8.0	4.8
	C0C1 stab	0.8	1.5	4.4	10.2	8.8	17.0					6.7	1.2
	Difference	− 1.3	− 12.8	− 11.9	− 9.5	2.2	− 6.7					− 1.3	− 3.6
10	Normal	0.4	5.6	11.2	16.0	6.8	20.0		3.0	5.1	6.2	5.5	7.4
	C0C1 stab	0.1	0.6	1.2	1.9	13.5	10.0			0.0	0.4	13.5	2.9
	Difference	− 0.3	− 5.0	− 10.0	− 14.1	6.7	− 10.0			− 5.1	− 5.8	8.0	− 4.5
Normal	Mean	7.6	13.6*	16.5*	18.3*	5.6	19.8*	15.4	12.2	10.2*	9.4*	6.8*	14.3*
	SD	6.6	5.9	5.6	6.0	1.5	5.2	13.6	9.7	8.0	8.0	2.6	7.7
C01 Stab	Mean	3.3	5.0*	5.5*	6.9*	7.2	11.5*		2.2	3.3*	3.9*	9.8*	6.6*
	SD	3.9	4.5	4.0	4.8	5.3	4.3		1.5	3.3	3.5	4.4	3.5
Diff	Mean	− 3.8	− 8.5	− 10.8	− 10.9	1.6	− 8.2		− 11.7	− 7.8	− 9.4	3.0	− 7.7
	SD	6.7	5.6	4.7	4.8	5.1	4.1		15.8	7.9	7.2	3.8	6.1

The table shows the values for all the specimens before (normal) and after (C0C1 stab) the stabilization of C0–C1. The means and standard deviations for each analyzed force and ROM Max are presented at the last rows of the table.

N, Newtons; F max, applied force at end range of motion; ROM max, end range of motion; SD, standard deviation.

*Statistical significance *p* < 0.05 values indicated in bold.

During upper cervical flexion, the end ROM without C0–C1 stabilization was 19.8° ± 5.2°, with an average maximum force of 5.6 N ± 1.5 N. All specimens demonstrate a reduction in flexion after C0–C1 stabilization (averaged end ROM of 11.5° ± 4.3°). The average maximum force was 7.2 N ± 5.3 N. Following C0–C1 stabilization, flexion ROM decreased during all standardized forces.

During upper cervical extension, the end ROM without C0–C1 stabilization was 14.3° ± 7.7°, with an average maximum force of 6.8 N ± 2.6 N. All specimens demonstrated a reduction in extension ROM following the stabilization of C0–C1 (6.6° ± 3.5° with an average maximum force of 9.8 N ± 4.4 N) during all standardized forces except specimen 7. However, if flexion and extension ROM are grouped together, specimen 7 demonstrated a reduction in ROM of 17.0° following C0–C1 stabilization.

### Upper cervical frontal plane mobility

**Figure 4:**
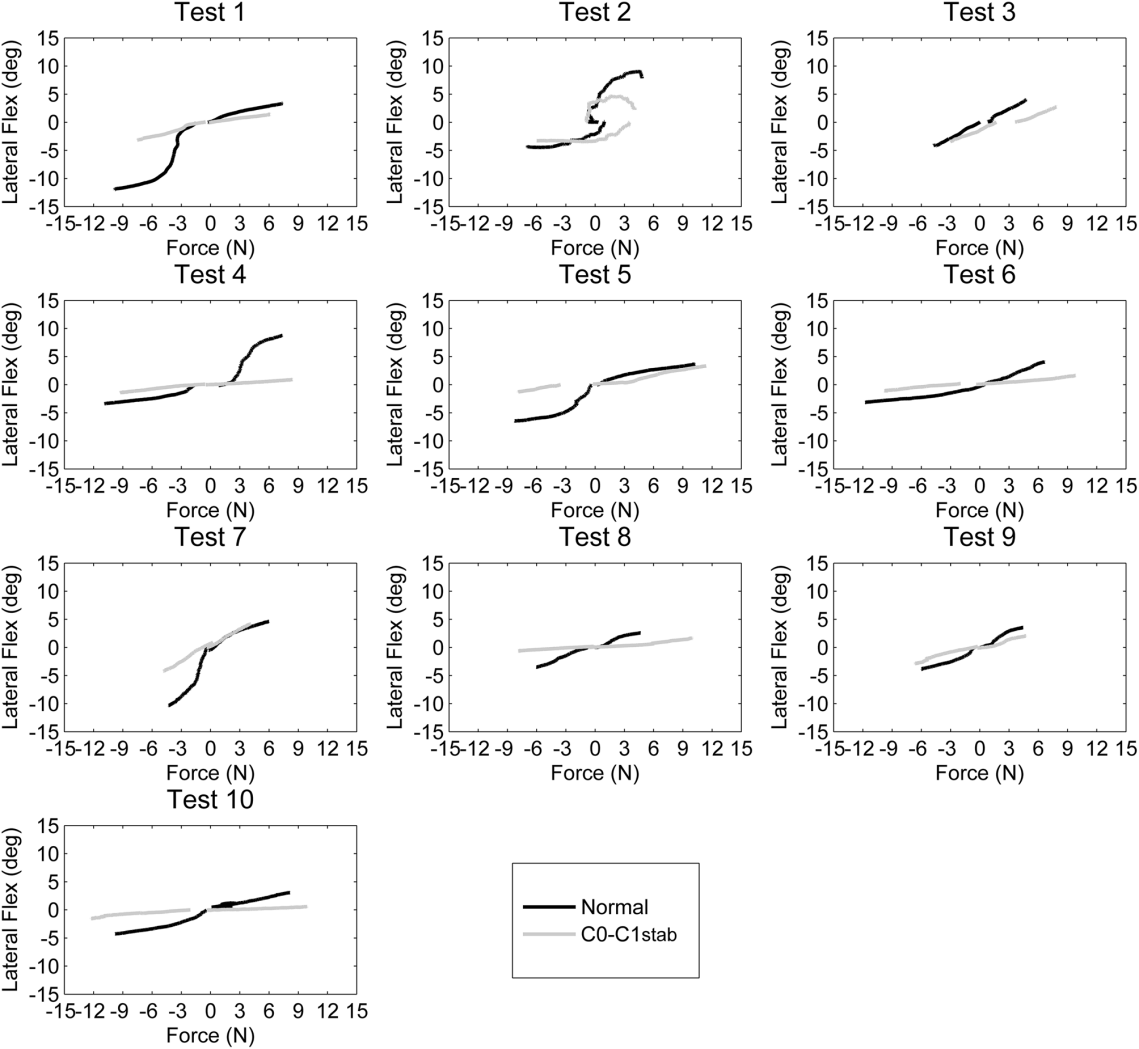
Forces required for left (negative values) and right lateral flexion (positive values) during the full range of motion in the 10 specimens: normal and with C0–C1 stabilization.

**Figure 5:**
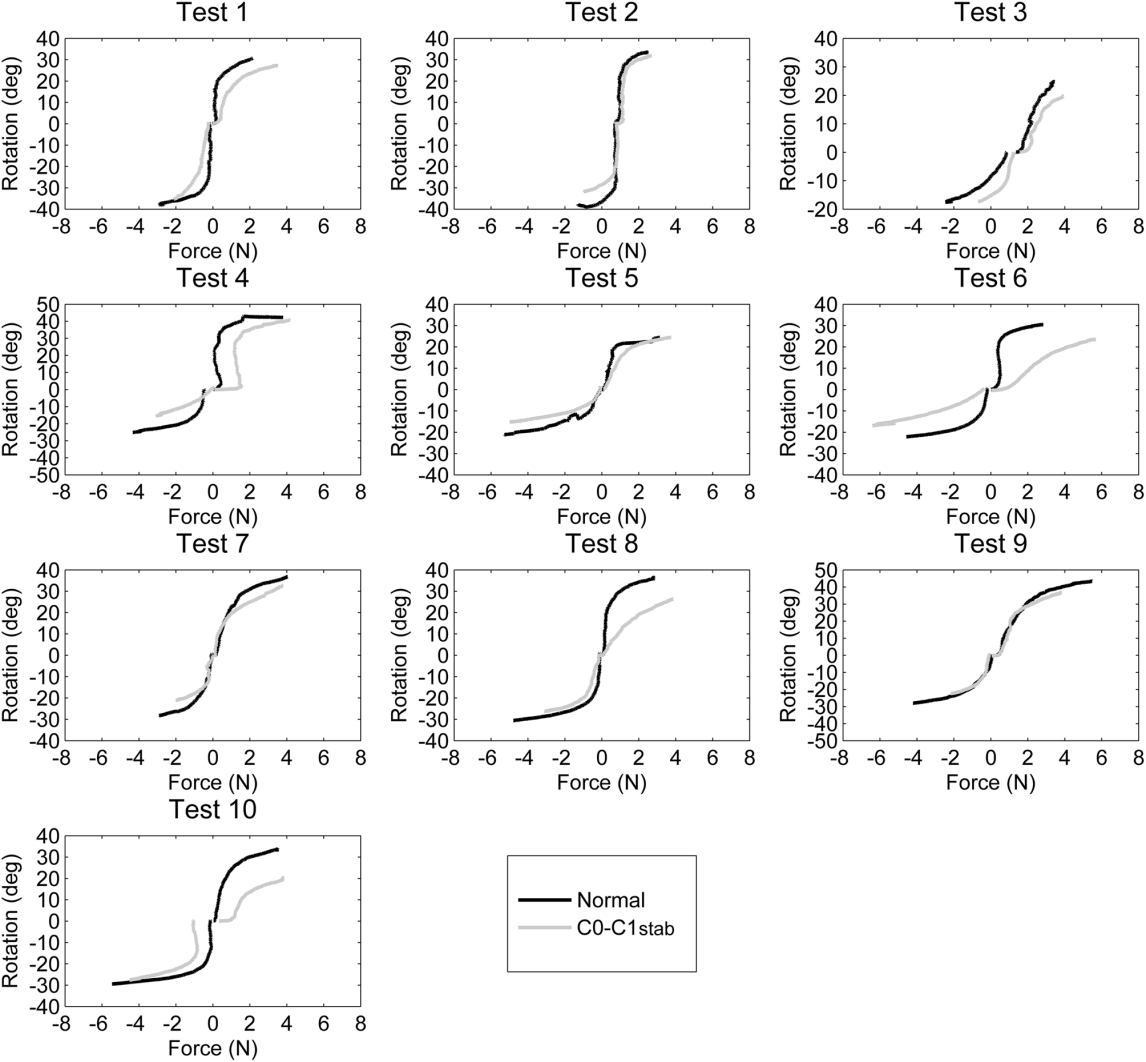
Forces required for left (negative values) and right axial rotation (positive values) during the full range of motion in the 10 specimens: normal and with C0–C1 stabilization.

Figures 4 and 5 represent the force applied and the resultant movement for lateral flexion and axial rotation, respectively. In Fig. 4, positive values indicate right lateral flexion, and negative values mean left lateral flexion. In Fig. 5, positive values indicate right axial rotation, and negative values indicate left axial rotation. Tables 3 and 4 contain the ROM for lateral flexion and rotation at 1 N, 2 N, 3 N, and 4 N, as well as the force applied to achieve maximum ROM (without and with C0–C1 stabilization). The ROM values from flexion to the zero position have not been included (empty boxes) in the table.

**Table 3:** Lateral flexion in degrees for the force values of 1, 2, 3, and 4 N during the motion, and the maximum force (F. Max) with its range of motion (ROM Max).

Test		Right lateral flexion (degrees)						Left lateral flexion (degrees)					
		Force					ROM Max	Force					ROM Max
		1 N	2 N	3 N	4 N	F. Max		1 N	2 N	3 N	4 N	F. Max	
1	Normal	0.9	1.5	1.9	2.3	7.4	3.4		0.7	1.7	7.2	9.9	12.1
	C0C1 stab	0.2	0.5	0.8	0.9	6.1	1.4	0.1	0.3	1.2	1.7	7.5	3.2
	Difference	− 0.7	− 1.0	− 1.1	− 1.4	− 1.3	− 2.0		− 0.4	− 0.5	− 5.5	− 2.4	− 8.9
2	Normal	6.0	7.7	8.5	8.9	4.8	9.0	2.7	3.2	3.9	4.2	7.0	4.5
	C0C1 stab	3.8	4.5	4.1	2.4	4.1	4.7	3.4	3.4	3.3	3.3	6.0	3.4
	Difference	− 2.2	− 3.2	− 4.4	− 6.5	− 0.7	− 4.3	0.7	0.2	− 0.6	− 0.9	− 1.0	− 1.1
3	Normal	0.2	1.5	2.4	3.2	4.7	4.1	0.8	1.9	2.9	3.7	4.8	4.1
	C0C1 stab				0.2	7.8	2.7	2.1	2.7	3.4		3.0	3.4
	Difference				− 3.0	3.1	− 1.4	1.3	0.8	0.5		− 1.8	− 0.7
4	Normal	0.0	0.3	2.6	5.4	7.4	8.8		0.5	1.4	1.8	10.9	3.4
	C0C1 stab	0.1	0.2	0.3	0.4	8.4	0.9		0.1	0.2	0.3	9.3	1.4
	Difference	0.1	− 0.1	− 2.3	− 5.0	1.0	− 7.9		− 0.4	− 1.2	− 1.5	− 1.6	− 2.0
5	Normal	0.7	1.3	1.7	2.1	10.2	3.7	1.7	3.3	4.8	5.4	8.3	6.5
	C0C1 stab	0.2	0.3	0.4	0.6	11.4	3.4				0.2	7.9	1.3
	Difference	− 0.5	− 1.0	− 1.3	− 1.5	1.2	− 0.3				− 5.2	− 0.4	− 5.2
6	Normal	0.5	0.9	1.4	2.3	6.7	4.1	0.8	1.1	1.5	1.8	11.8	3.1
	C0C1 stab	0.2	0.3	0.5	0.6	9.8	1.7				0.1	9.8	1.1
	Difference	− 0.3	− 0.6	− 0.9	− 1.7	3.1	− 2.4				− 1.7	− 2.0	− 2.0
7	Normal	1.0	2.2	3.0	3.6	6.0	4.6	3.3	7.0	8.7	10.0	4.3	10.4
	C0C1 stab	1.2	2.3	3.2	4.1	4.1	4.2	0.2	1.3	2.5	3.6	4.8	4.3
	Difference	0.2	0.1	0.2	0.5	− 1.9	− 0.4	− 3.1	− 5.7	− 6.2	− 6.4	0.5	− 6.1
8	Normal	0.4	1.5	2.1	2.4	4.7	2.6	0.2	0.7	1.5	2.3	6.0	3.5
	C0C1 stab	0.1	0.2	0.3	0.4	9.9	1.7			0.1	0.2	7.9	0.7
	Difference	− 0.3	− 1.3	− 1.8	− 2.0	5.2	− 0.9			− 1.4	− 2.1	1.9	− 2.8
9	Normal	0.4	1.9	2.9	3.4	4.5	3.5	0.9	1.9	2.5	3.0	6.0	3.9
	C0C1 stab	0.1	0.6	1.4	1.8	4.7	2.1	0.1	0.5	0.8	1.3	6.6	2.9
	Difference	− 0.3	− 1.3	− 1.5	− 1.6	0.2	− 1.4	− 0.8	− 1.4	− 1.7	− 1.7	0.6	− 1.0
10	Normal	0.7	1.2	1.3	1.6	8.1	3.1	0.8	1.6	2.2	2.7	9.8	4.3
	C0C1 stab	0.0	0.1	0.1	0.1	9.9	0.6			0.1	0.2	12.2	1.5
	Difference	− 0.7	− 1.1	− 1.2	− 1.5	1.8	− 2.5			− 2.1	− 2.5	2.4	− 2.8
Normal	Mean	1.1*	2.0*	2.8*	3.5*	6.5	4.7*	1.4	2.2	3.1*	4.2*	7.9	5.6*
	SD	1.8	2.1	2.1	2.2	1.9	2.3	1.1	2.0	2.3	2.6	2.6	3.2
C0C1 Stab	Mean	0.7*	1.0*	1.2*	1.1*	7.6	2.3*	1.2	1.4	1.5*	1.2*	7.5	2.3*
	SD	1.2	1.5	1.4	1.3	2.7	1.4	1.5	1.4	1.4	1.4	2.6	1.2
Diff	Mean	− 0.5	− 1.1	− 1.6	− 2.4	1.2	− 2.4	− 0.5	− 1.2	− 1.7	− 3.1	− 0.4	− 3.3
	SD	0.7	0.9	1.3	2.0	2.2	2.3	2.0	2.3	2.0	2.1	1.7	2.7

The table shows the values for all the specimens before (normal) and after (C0C1 stab) the stabilization of C0–C1. The means and standard deviations for each analyzed force and ROM Max are presented at the last rows of the table.

N, Newtons; F max, applied force at end range of motion; ROM max, end range of motion; SD, standard deviation.

*Statistical significance *p* < 0.05 values indicated in bold.

**Table 4:** Rotation in degrees for the force values of 1, 2, 3, and 4 N during the motion, and the maximum force (F. Max) with its range of motion (ROM Max).

Test		Right rotation (degrees)						Left rotation (degrees)					
		Force					ROM Max	Force					ROM Max
		1 N	2 N	3 N	4 N	F. Max		1 N	2 N	3 N	4 N	F. Max	
1	Normal	26.1	30.0			2.2	30.8	33.6	36.0			2.9	38.0
	C0C1 stab	16.6	23.5	26.3		3.5	27.3	26.1	34.9			2.1	41.9
	Difference	− 9.5	− 6.5			1.3	− 3.5	− 7.5	− 1.1			− 0.8	3.9
2	Normal	5.7	32.6			2.5	33.5	38.5				1.3	39.0
	C0C1 stab	0.4	30.0			2.7	31.9					1.0	31.9
	Difference	− 5.3	− 2.6			0.2	− 1.6					− 0.3	− 7.1
3	Normal	0.0	7.5	21.8		3.5	24.8	13.6	16.4			2.5	17.6
	C0C1 stab		1.0	15.9		3.9	19.8					0.7	17.3
	Difference		− 6.5	− 5.9		0.4	− 5.0					− 1.8	− 0.3
4	Normal	38.0	42.7	42.5		3.8	43.9	15.6	20.7	22.7	24.4	4.3	25.4
	C0C1 stab	0.4	34.9	38.3	40.3	4.2	40.9	7.1	11.4	15.2		3.1	15.7
	Difference	− 37.6	− 7.8	− 4.2		0.4	− 3.0	− 8.5	− 9.3	− 7.5		− 1.2	− 9.7
5	Normal	21.3	22.1	24.2		3.1	24.6	12.2	14.5	17.8	19.4	5.3	21.1
	C0C1 stab	15.0	21.3	23.3		3.7	24.5	7.9	11.0	12.6	14.0	5.0	15.2
	Difference	− 6.3	− 0.8	− 0.9		0.6	− 0.1	− 4.3	− 3.5	− 5.2	− 5.4	− 0.3	− 5.9
6	Normal	26.7	29.2			2.8	30.5	15.1	18.8	20.4	21.6	4.6	22.1
	C0C1 stab	1.3	8.9	15.3	19.5	5.7	23.6	3.5	7.5	10.4	12.7	6.4	17.0
	Difference	− 25.4	− 20.3			2.9	− 6.9	− 11.6	− 11.3	− 10.0	− 8.9	1.8	− 5.2
7	Normal	22.7	31.0	34.1	36.7	4.0	36.9	21.5	26.3			2.9	28.4
	C0C1 stab	19.6	24.9	29.4		3.8	32.8	18.0				2.0	21.1
	Difference	− 3.1	− 6.1	− 4.7		− 0.2	− 4.1	− 3.5				− 0.9	− 7.3
8	Normal	30.0	34.3			2.9	36.5	23.2	26.5	28.4	29.6	4.8	30.8
	C0C1 Stab	11.4	19.2	23.4		3.9	26.5	19.8	24.3	26.0		3.1	26.3
	Difference	− 18.6	− 15.1			1.0	− 10.0	− 3.4	− 2.2	− 2.4		− 1.7	− 4.5
9	Normal	16.1	31.1	37.6	40.4	5.5	43.5	18.9	23.7	26.0	27.7	4.2	28.0
	C0C1 Stab	18.7	28.9	33.7		3.8	36.8	18.3	22.0			2.2	22.5
	Difference	2.6	− 2.2	− 3.9		− 1.7	− 6.7	− 0.6	− 1.7			− 2.0	− 5.5
10	Normal	24.3	30.1	32.5		3.6	33.7	23.4	26.0	27.3	28.2	5.5	29.5
	C0C1 stab	0.8	13.6	17.5		3.8	20.5	16.9	22.5	24.9	26.7	4.5	27.6
	Difference	− 23.5	− 16.5	− 15.0		0.2	− 13.2	− 6.5	− 3.5	− 2.4	− 1.5	− 1.0	− 1.9
Normal	Mean	**21.1**	29.1*	32.1*	38.6	3.4	33.9*	21.6*	23.2*	23.8*	25.2	3.8	28.0*
	SD	11.3	9.1	7.9	2.6	0.9	6.7	8.6	6.5	4.2	4.0	1.4	6.9
C0C1 stab	Mean	9.4*	20.6*	24.8*	29.9	3.9	28.5*	14.7*	19.1*	17.8*	17.8	3.0	23.7*
	SD	8.5	10.3	8.0	14.7	0.7	7.0	7.7	9.6	7.2	7.7	1.8	8.5
Diff	Mean	− 14.1	− 8.4	− 5.8		0.5	− 5.4	− 5.7	− 4.7	− 5.5	− 5.3	− 0.8	− 4.4
	SD	13.0	6.6	4.8		1.2	3.9	3.5	4.0	3.3	3.7	1.1	3.9

The table shows the values for all the specimens before (normal) and after (C0C1 stab) the stabilization of C0–C1. The means and standard deviations for each analyzed force and ROM Max are presented at the last rows of the table.

N: Newtons; F max: applied force at end range of motion; ROM max: end range of motion; SD: standard deviation.

*Statistical significance *p* < 0.05 values indicated in bold.

During upper cervical right lateral flexion, the end ROM without C0–C1 stabilization was 4.7° ± 2.3°, with an average maximum force of 6.5 N ± 1.9 N. Following C0–C1 stabilization, all specimens demonstrated a reduction in ROM (2.3° ± 1.4°) at all standardized forces with an average maximum force of 7.6 N ± 2.7 N.

During upper cervical left lateral flexion, the end ROM without C0–C1 stabilization was 5.6° ± 3.2° with a maximum force of 7.9 N ± 2.6 N. Following C0–C1 stabilization all specimens demonstrated a reduction in ROM (2.3° ± 1.2°) at all standardized forces with an average maximum force of 7.5 N ± 2.6 N.

### Upper cervical transverse plane mobility

During upper cervical right axial rotation, the end ROM without C0–C1 stabilization was 33.9° ± 6.7°, with an average maximum force of 3.4 N ± 0.9 N. Following C0–C1 stabilization, all specimens demonstrated a reduction in ROM (28.5° ± 7.0°) at all standardized forces with an average maximum force of 3.9 N ± 0.7 N.

During upper cervical left axial rotation, the average end ROM without C0–C1 stabilization was 28.0° ± 6.9°, with an average maximum force of 3.8 N ± 1.4 N. All specimens demonstrated a reduction in left rotation ROM following the stabilization of C0–C1 (23.7° ± 8.5° with an average maximum force of 3.0 N ± 1.8 N) during all standardized forces except specimen 1, which had a maximum force 0.8 N lower with C0–C1 stabilization versus non-stabilization.

Table 5 shows the statistical significance of the maximal force applied and ROM at different standardized forces and end-range for non-stabilized and C0–C1 stabilization configurations in the three cardinal planes. At the end ROM, all directions of movements showed a statistically significant reduction of movement with C0–C1 stabilization. There were no statistical differences in the maximal forces applied without and with stabilization of C0–C1 in all directions except for extension in which more force was applied with stabilization of C0–C1 (*p* = 0.03).

**Table 5:** Statistical significance of the maximal force applied and range of motion (degrees) at different standardized forces and end-range for normal and stabilized C0–C1 configurations in flexion, extension, right lateral flexion, left lateral flexion, right axial rotation, and left axial rotation.

	Flexion		Extension		Right lateral flexion		Left lateral flexion		Right rotation		Left rotation	
	Mean ± SD	*p* value	Mean ± SD	*p* value	Mean ± SD	*p* value	Mean ± SD	*p* value	Mean ± SD	*p* value	Mean ± SD	*p* value
1N_Normal	7.6 ± 6.6	0.110	15.4 ± 13.6	–	1.1 ± 1.8	**0.021**	1.4 ± 1.1	0.715	21.1 ± 11.3	**0.011**	21.6 ± 8.6	**0.012**
1N_Stabilized	3.3 ± 3.9		-		0.7 ± 1.2		1.2 ± 1.5		9.4 ± 8.5		14.7 ± 7.7	
2N_Normal	13.6 ± 5.9	**0.008**	12.2 ± 9.7	0.180	2.0 ± 2.1	**0.011**	2.2 ± 2.0	0.249	29.1 ± 9.1	**0.005**	23.2 ± 6.5	**0.018**
2N_Stabilized	5.0 ± 4.5		2.2 ± 1.5		1.0 ± 1.5		1.4 ± 1.4		20.6 ± 10.3		19.1 ± 9.6	
3N_Normal	16.5 ± 5.6	**0.012**	10.2 ± 8.0	**0.043**	2.8 ± 2.1	**0.011**	3.1 ± 2.3	**0.021**	32.1 ± 7.9	**0.028**	23.8 ± 4.2	**0.043**
3N_Stabilized	5.5 ± 4.0		3.3 ± 3.3		1.2 ± 1.4		1.5 ± 1.4		24.8 ± 8.0		17.8 ± 7.2	
4N_Normal	18.3 ± 6.0	**0.028**	9.4 ± 8.0	**0.028**	3.5 ± 2.2	**0.007**	4.2 ± 2.6	**0.008**	38.6 ± 2.6	–	25.2 ± 4.0	0.109
4N_Stabilized	6.9 ± 4.8		3.9 ± 3.5		1.1 ± 1.3		1.2 ± 1.4		29.9 ± 14.7		17.8 ± 7.7	
NMax_Normal	5.6 ± 1.5	0.721	6.8 ± 2.6	**0.037**	6.5 ± 1.9	0.169	7.9 ± 2.6	0.575	3.4 ± 0.9	0.093	3.8 ± 1.4	0.059
NMax_Stabilized	7.2 ± 5.3		9.8 ± 4.4		7.6 ± 2.7		7.5 ± 2.6		3.9 ± 0.7		3.0 ± 1.8	
ROMMax_Normal	19.8 ± 5.2	**0.005**	14.3 ± 7.7	**0.007**	4.7 ± 2.3	**0.005**	5.6 ± 3.2	**0.005**	33.9 ± 6.7	**0.005**	28.0 ± 6.9	**0.013**
ROMMax_Stabilizd	11.5 ± 4.3		6.6 ± 3.5		2.3 ± 1.4		2.3 ± 1.2		28.5 ± 7.0		23.7 ± 8.5	

N: Newtons; F max: applied force at end range of motion; ROM max: end range of motion; SD: standard deviation.

*p* values in bold showed statistical significance (*p* < 0.05).

## Discussion

To our knowledge, this is the first biomechanical study that analyzes the role of C0–C1 restriction of movement on UCS kinematics (C0–C1 and C1–C2). Screw stabilization of C0 achieved a consistent reduction of mobility in C0–C1, especially in the transverse plane.

The results of this study show that C0–C1 stabilization results in a statistically significant reduction of the ROM in the three cardinal planes. Stabilization of C0–C1 resulted in a reduction of 46.9%, 55.3%, and 15.6% in the upper cervical motion in the sagittal, frontal, and transverse plane, respectively. Also, when considering ROM with standardized forces, the stabilization of C0–C1 produced lower ROM than the non-stabilized configuration.

Sample size, age-related degenerative changes, and frequent upper cervical anatomy variations should also be considered when analyzing our results^28^. For example, anatomical variations for alar ligaments, including ligament orientation from dens to the occiput (craniocaudal, horizontal, or caudocranial)^29^, variability in the origin of the ligaments on the odontoid process, and an inconsistent atlantal portion of the alar ligament^30^ have been described in the literature. This inter-individual variability is likely to lead to differences in our results in the sagittal plane (Table 2), frontal plane (Table 3), and transverse plane (Table 4). For example, some specimens did not show any change during axial rotation following C0–C1 stabilization. In contrast, others showed a reduction of up to 74% during axial rotation, demonstrating a very relevant role of C0–C1 in the upper cervical rotation. Inter-individual variations are also likely to lead to differences in results.

In the sagittal plane, upper cervical movement without stabilization was 34.1° in our specimens (19.8° in flexion and 14.3° in extension). These results are similar to previously reported values of 15–25° in flexion^2^ and reduced compared to the 46° reported by Ernst et al. (2015) in a sample of patients with non-specific cervicalgia. The results of Ernst et al. (2015) are not directly comparable to this study since they used an in vivo design and active motion without stabilization of C2^31^.

With C0–C1 stabilization, there was a reduction of 16.0° in the sagittal plane movement. This value is similar to the average C0–C1 flexion–extension of 14–15° in most of the studies^2^. The remaining 18.1° measured during C0–C1 stabilization (C1–C2 and remaining C0–C1 contribution after stabilization) are within the 10–21° reported in the literature^2^.

Using the Frankfort plane as the zero position, C0–C1 accounted for 41.9% of the upper cervical flexion (or in other words: after C0–C1 stabilization, the 58.1% of the normal C0-C2 ROM was obtained). Similarly, Chancey et al. (2007) reported that C0–C1 produced 41–45% of UCS flexion. In our sample, C0–C1 produced at least 53.8% of the upper cervical extension (after C0–C1 stabilization, 46.2% of the normal C0-C2 ROM was obtained). This value differs from the reported 69–71% of the upper cervical extension occurring in C0–C1^4^. However, the C0–C1 ROM in our sample should be larger since the screw stabilization did not abolish C0–C1 ROM totally. The remaining UCS ROM after C0–C1 stabilization found within this study supports the importance of the C1–C2 segment during upper cervical flexion and extension.

In the frontal plane, upper cervical movement without stabilization was 4.7° in right lateral flexion and 5.6° in left lateral flexion. Frontal plane movements are rarely reported in the literature and are considered by some to be non-physiological movements of the atlanto-occipital joints^2^. However, motion in the frontal plane could vary between individuals. Some of the specimens in this study (1 and 7) moved approximately 15° in the frontal plane. Frequently observed anatomical variations in the upper cervical spine could explain this specimen specific movement ^32^.

As an average, it seems that both C0–C1 and C1–C2 participate similarly in the lateral flexion movement. During this study, C0–C1 stabilization produced 51.3% reduction in right lateral flexion and 58.0% in left lateral flexion.

In the transverse plane, upper cervical movement without stabilization was 61.9° (33.9° and 28.0° for right and left axial rotation, respectively). The results for upper cervical rotation are lower than the in vitro studies with reported reference values from 66.6°^33^ to 92.4°^34^. This is likely due to methodological differences between this study and those previously published.

C0–C1 stabilization reduced transverse plane movement 9.8° (15.7% of the upper cervical ROM in this plane). The limitation of upper cervical axial rotation of C0–C1 in our study is similar to the in vitro studies of Panjabi et al. (1988)^34^ (14.8%) and Panjabi et al. (2001)^35^ (14.86%) but higher than other in vivo studies with active movements (2.4–8.9%)^9,10,36–39^. In general, it seems to be a lack of data regarding the contribution of C0–C1 to upper cervical axial rotation in the literature, and in fact, some authors even disregard it ^7,9,11–13,40^. Boszczyk et al. (2012) concluded that only considering C1–C2 arthrokinematics do not explain the tolerance of the alar ligaments at the maximum of 40° of UCS rotation^12^. The findings of Boszczyk et al. suggest that C0–C1 could play a more relevant function during passive UCS rotation. In our study, C0–C1 stabilization reduced upper cervical rotatory ROM more than the reported C0–C1 range in the literature in the same direction as UCS rotation (2.5° ± 1°)^10^ or even in the opposite direction as UCS rotation (− 1°)^41,42^ at the end of the upper cervical rotation. Also, at 1 N, 2 N and 3 N mobilization load, the UCS rotation with C0–C1 stabilization was significantly lower than in the non-stabilized condition. The C0–C1 restriction of movement may have had an influence on alar ligament tightening. Further support for the contribution of C0–C1 during UCS axial rotation is reported clinically showing an increase of C1–C2 ROM following C0–C1 mobilization^15–19^, although scientific evidence about the specific segmental effect in C0–C1 and not in adjacent segments of C0–C1 translatoric mobilization is needed.

The alar ligaments are considered a primary restraint to axial rotation^22,43,44^. Findings from this study indicate there is a reduction of the upper cervical axial rotation ROM and increased forces when C0–C1 is stabilized compared to non-stabilization. This increase of resistance in upper cervical axial rotation with C0–C1 stabilization could mean that C0–C1 kinematics are related to the tightening of the alar ligaments and indirectly, to the upper cervical and C1–C2 ROM in the transverse plane. In fact, research investigating the impact of the alar ligament on upper cervical axial rotation indicate that alar ligament transection increases C0–C1 axial rotation by 30%^37^.

The data from our study provides insight into the effect of surgical applications of treating C0–C1 dislocation via C0–C1 transcondylar screw techniques^45–47^. We observed a ROM reduction after C0–C1 stabilization in each plane as happens with the surgical insertion of transarticular screws. It is known that adding a structural graft may further improve the amount of stability in the C0–C1 segment^46^. However, the results of this study are not directly comparable to the typical surgical procedure because of the different fixating method and the different entry point and screw’s trajectory from the in vivo techniques. Even with these differences in stabilization methods, this study provides valuable 3D motion and load information during a simulated manual clinical procedure used to examine upper cervical kinematics.

Other limitations of the present study relate to the mobilization procedure. The methodology used was original and specific to the objectives but challenging to compare with prior studies. The in vitro design allowed the stabilization of C2 as a fixed point for movement reference. The mobilization force was manually applied to simulate a clinical and physiological procedure in comparison to loading devices. Inducing the mobilization manually challenges the repeatability in terms of direction and magnitude of the loads. However, after the experimental testing, the study compared certain force values (1 N, 2 N, 3 N, and 4 N) in each of the planes in both conditions. Physiological motion can also be produced by machines^34^. However, intersegmental movement outside the primary plane of motion (coupled motions) has also been reported in experimental testing using machines loading in one anatomical plane^48^. Also, the structures dissected before the applied movements^49^ may also influence the results.

This in vitro study, showed a reduction in all cardinal plane motions following stabilization of C0–C1. During transverse plane motion, C0–C1 stabilization reduced upper cervical rotation by 15%, a higher rate than expected, considering the reported C0–C1 rotational range of movement in the literature. In addition, the increase of resistance in upper cervical axial rotation with C0–C1 stabilization could mean that C0–C1 kinematics could be related to the tightening of the alar ligaments.
